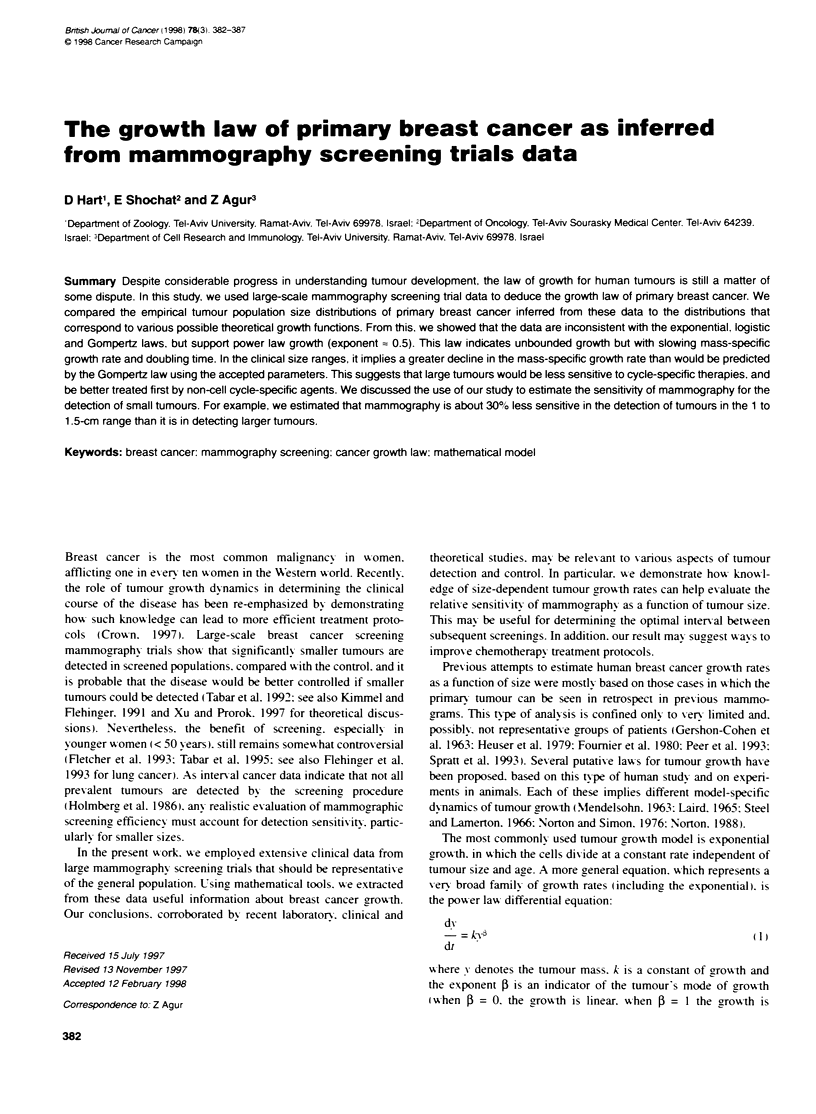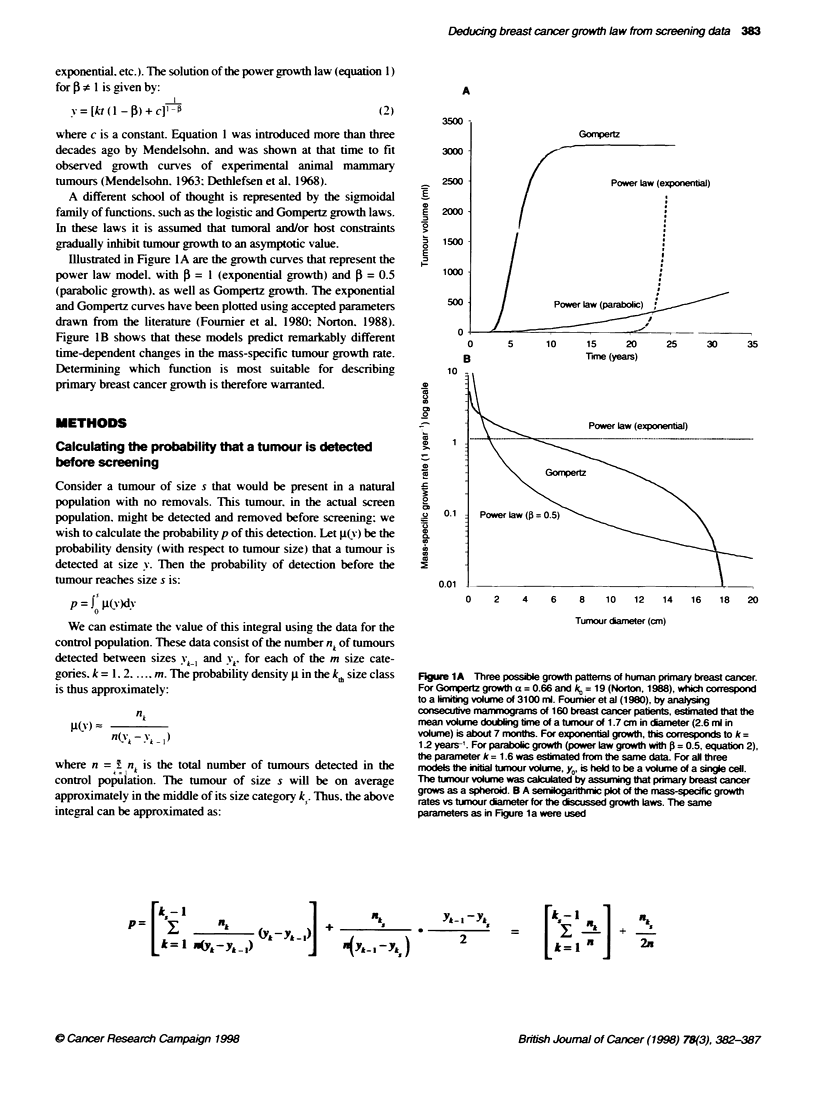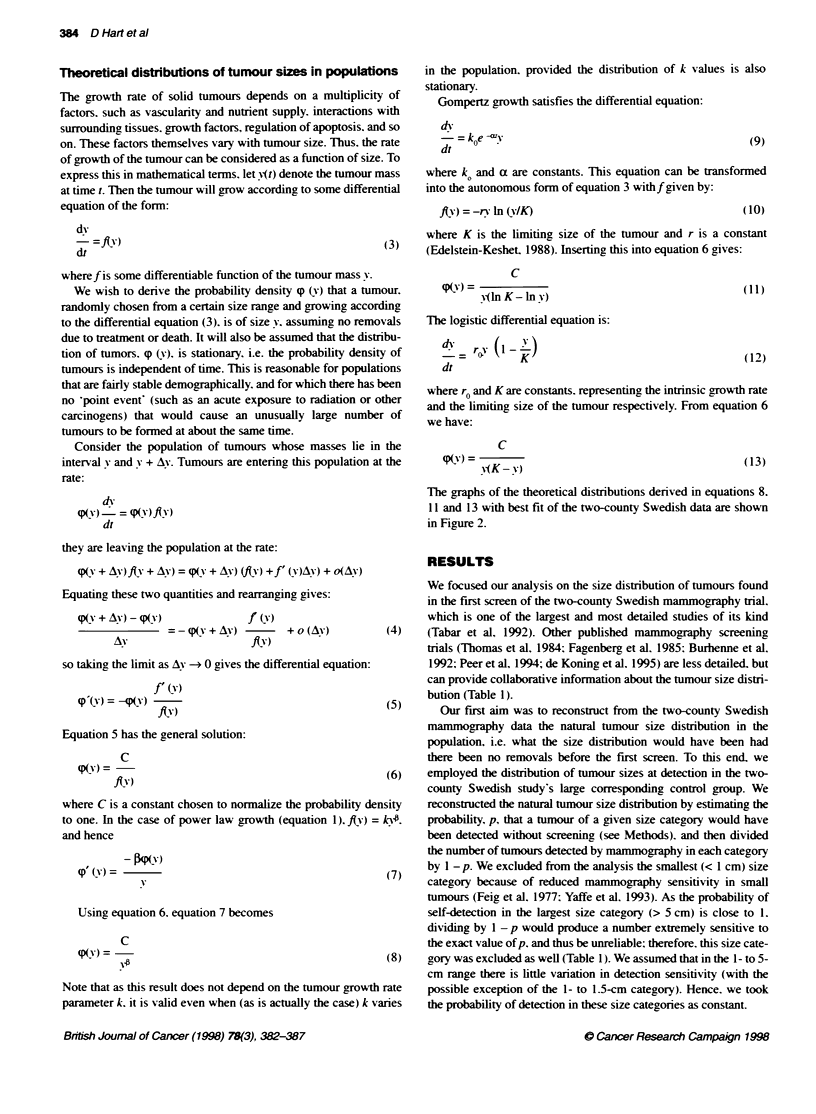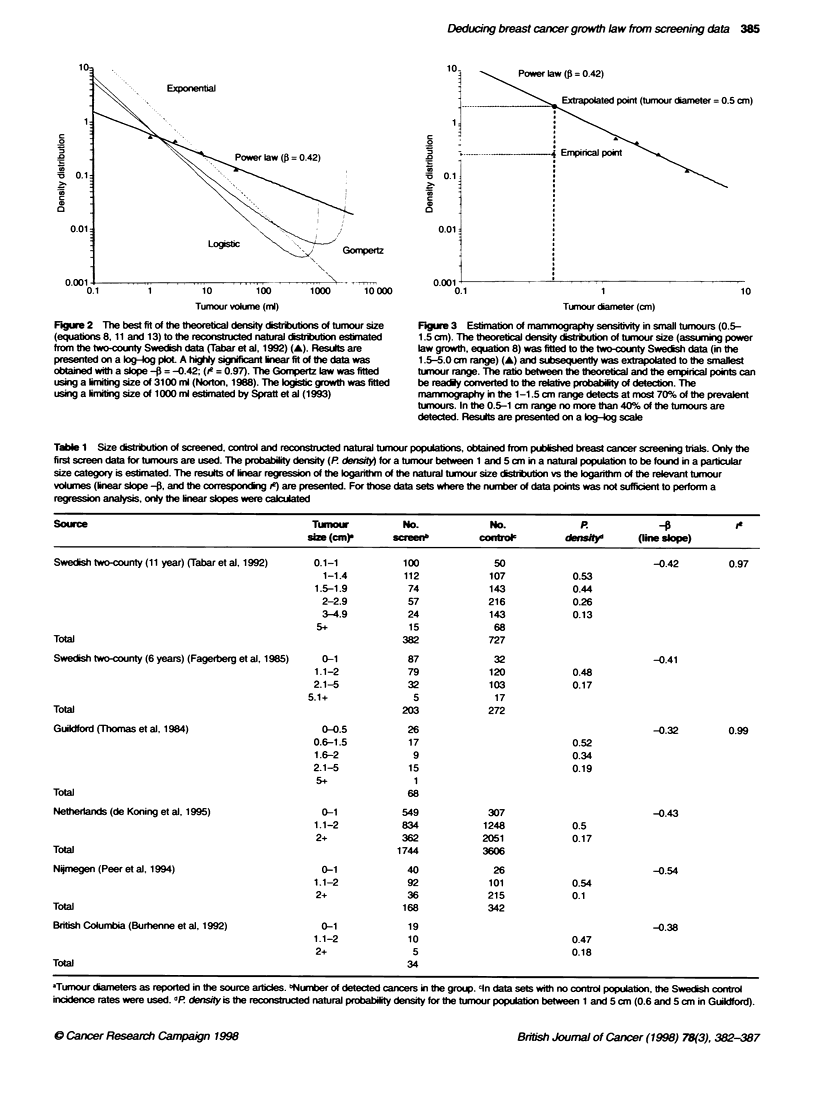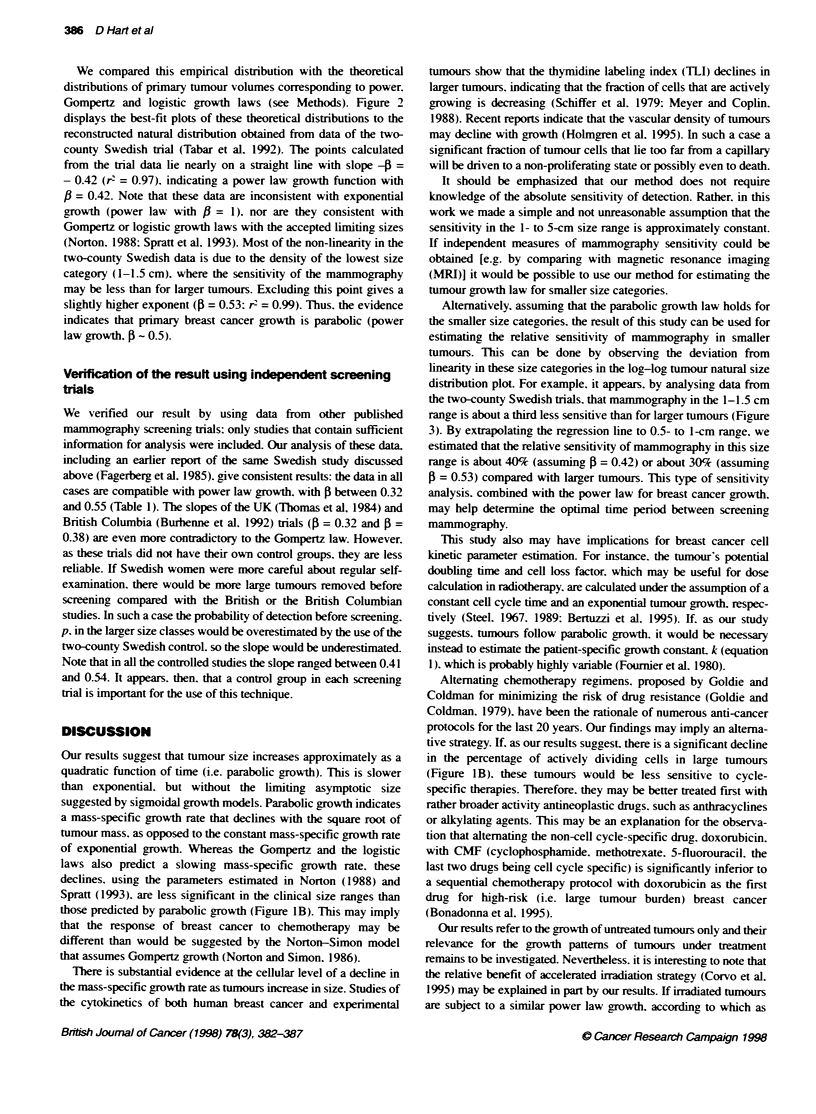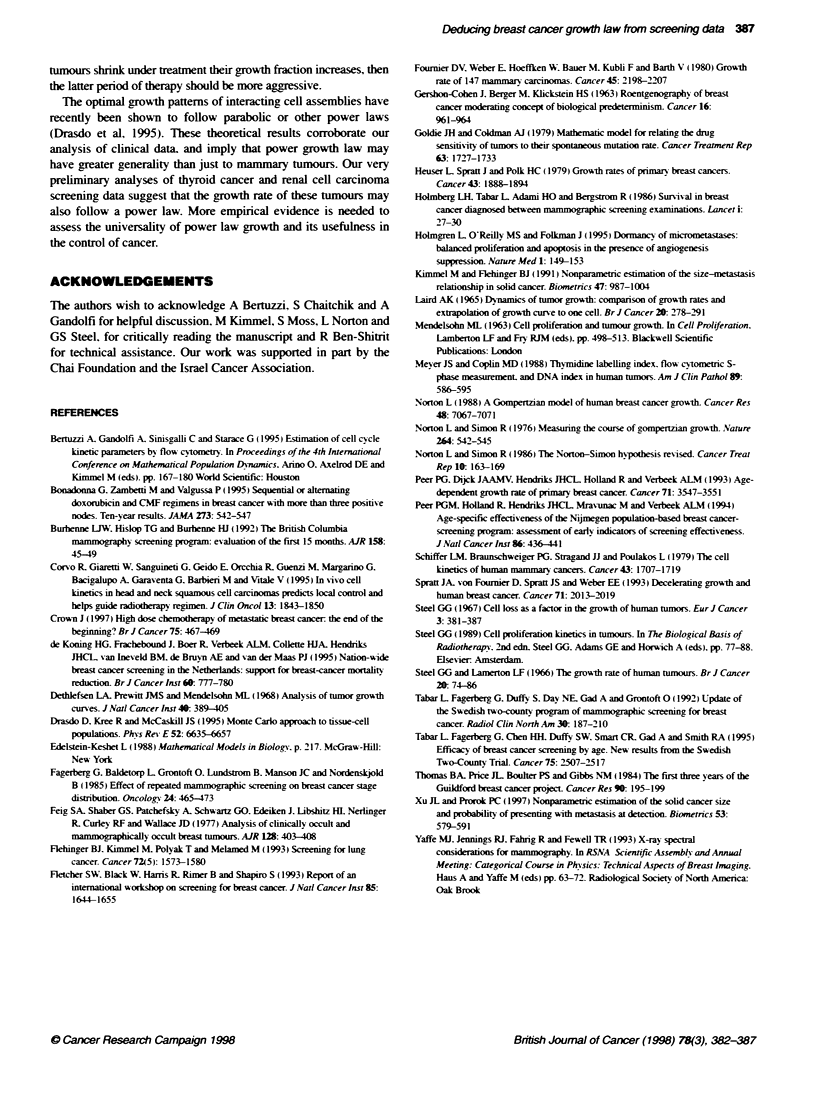# The growth law of primary breast cancer as inferred from mammography screening trials data.

**DOI:** 10.1038/bjc.1998.503

**Published:** 1998-08

**Authors:** D. Hart, E. Shochat, Z. Agur

**Affiliations:** Department of Zoology, Tel-Aviv University, Ramat-Aviv, Israel.

## Abstract

Despite considerable progress in understanding tumour development, the law of growth for human tumours is still a matter of some dispute. In this study, we used large-scale mammography screening trial data to deduce the growth law of primary breast cancer. We compared the empirical tumour population size distributions of primary breast cancer inferred from these data to the distributions that correspond to various possible theoretical growth functions. From this, we showed that the data are inconsistent with the exponential, logistic and Gompertz laws, but support power law growth (exponent approximately 0.5). This law indicates unbounded growth but with slowing mass-specific growth rate and doubling time. In the clinical size ranges, it implies a greater decline in the mass-specific growth rate than would be predicted by the Gompertz law using the accepted parameters. This suggests that large tumours would be less sensitive to cycle-specific therapies, and be better treated first by non-cell cycle-specific agents. We discussed the use of our study to estimate the sensitivity of mammography for the detection of small tumours. For example, we estimated that mammography is about 30% less sensitive in the detection of tumours in the 1 to 1.5-cm range than it is in detecting larger tumours.


British Journal of Cancer (1998) 78(3), 382–387
© 1998 Cancer Research Campaign

### The growth law of primary breast cancer as inferred from mammography screening trials data

**D Hart[1], E Shochat[2] and Z Agur[3]**

[1]Department of Zoology, Tel-Aviv University, Ramat-Aviv, Tel-Aviv 69978, Israel; [2]Department of Oncology, Tel-Aviv Sourasky Medical Center, Tel-Aviv 64239, Israel; [3]Department of Cell Research and Immunology, Tel-Aviv University, Ramat-Aviv, Tel-Aviv 69978, Israel

**Summary** Despite considerable progress in understanding tumour development, the law of growth for human tumours is still a matter of some dispute. In this study, we used large-scale mammography screening trial data to deduce the growth law of primary breast cancer. We compared the empirical tumour population size distributions of primary breast cancer inferred from these data to the distributions that correspond to various possible theoretical growth functions. From this, we showed that the data are inconsistent with the exponential, logistic and Gompertz laws, but support power law growth (exponent ≈ 0.5). This law indicates unbounded growth but with slowing mass-specific growth rate and doubling time. In the clinical size ranges, it implies a greater decline in the mass-specific growth rate than would be predicted by the Gompertz law using the accepted parameters. This suggests that large tumours would be less sensitive to cycle-specific therapies, and be better treated first by non-cell cycle-specific agents. We discussed the use of our study to estimate the sensitivity of mammography for the detection of small tumours. For example, we estimated that mammography is about 30% less sensitive in the detection of tumours in the 1 to 1.5-cm range than it is in detecting larger tumours.

**Keywords:** breast cancer; mammography screening; cancer growth law; mathematical model

Breast cancer is the most common malignancy in women, afflicting one in every ten women in the Western world. Recently, the role of tumour growth dynamics in determining the clinical course of the disease has been re-emphasized by demonstrating how such knowledge can lead to more efficient treatment protocols (Crown, 1997). Large-scale breast cancer screening mammography trials show that significantly smaller tumours are detected in screened populations, compared with the control, and it is probable that the disease would be better controlled if smaller tumours could be detected (Tabar et al, 1992; see also Kimmel and Flehinger, 1991 and Xu and Prorok, 1997 for theoretical discussions). Nevertheless, the benefit of screening, especially in younger women (< 50 years), still remains somewhat controversial (Fletcher et al, 1993; Tabar et al, 1995; see also Flehinger et al, 1993 for lung cancer). As interval cancer data indicate that not all prevalent tumours are detected by the screening procedure (Holmberg et al, 1986), any realistic evaluation of mammographic screening efficiency must account for detection sensitivity, particularly for smaller sizes.

In the present work, we employed extensive clinical data from large mammography screening trials that should be representative of the general population. Using mathematical tools, we extracted from these data useful information about breast cancer growth. Our conclusions, corroborated by recent laboratory, clinical and theoretical studies, may be relevant to various aspects of tumour detection and control. In particular, we demonstrate how knowledge of size-dependent tumour growth rates can help evaluate the relative sensitivity of mammography as a function of tumour size. This may be useful for determining the optimal interval between subsequent screenings. In addition, our result may suggest ways to improve chemotherapy treatment protocols.

Previous attempts to estimate human breast cancer growth rates as a function of size were mostly based on those cases in which the primary tumour can be seen in retrospect in previous mammograms. This type of analysis is confined only to very limited and, possibly, not representative groups of patients (Gershon-Cohen et al, 1963; Heuser et al, 1979; Fournier et al, 1980; Peer et al, 1993; Spratt et al, 1993). Several putative laws for tumour growth have been proposed, based on this type of human study and on experiments in animals. Each of these implies different model-specific dynamics of tumour growth (Mendelsohn, 1963; Laird, 1965; Steel and Lamerton, 1966; Norton and Simon, 1976; Norton, 1988).

The most commonly used tumour growth model is exponential growth, in which the cells divide at a constant rate independent of tumour size and age. A more general equation, which represents a very broad family of growth rates (including the exponential), is the power law differential equation:

$$\frac{dy}{dt} = ky^{\beta} \qquad (1)$$

where $y$ denotes the tumour mass, $k$ is a constant of growth and the exponent $\beta$ is an indicator of the tumour's mode of growth (when $\beta = 0$, the growth is linear, when $\beta = 1$ the growth is

*Received 15 July 1997*
*Revised 13 November 1997*
*Accepted 12 February 1998*

*Correspondence to:* Z Agur exponential, etc.). The solution of the power growth law (equation 1) for $\beta \neq 1$ is given by:

$$y = [kt\,(1-\beta) + c]^{\frac{1}{1-\beta}} \qquad (2)$$

where $c$ is a constant. Equation 1 was introduced more than three decades ago by Mendelsohn, and was shown at that time to fit observed growth curves of experimental animal mammary tumours (Mendelsohn, 1963; Dethlefsen et al, 1968).

A different school of thought is represented by the sigmoidal family of functions, such as the logistic and Gompertz growth laws. In these laws it is assumed that tumoral and/or host constraints gradually inhibit tumour growth to an asymptotic value.

Illustrated in Figure 1A are the growth curves that represent the power law model, with $\beta = 1$ (exponential growth) and $\beta = 0.5$ (parabolic growth), as well as Gompertz growth. The exponential and Gompertz curves have been plotted using accepted parameters drawn from the literature (Fournier et al, 1980; Norton, 1988). Figure 1B shows that these models predict remarkably different time-dependent changes in the mass-specific tumour growth rate. Determining which function is most suitable for describing primary breast cancer growth is therefore warranted.

### METHODS

### Calculating the probability that a tumour is detected before screening

Consider a tumour of size $s$ that would be present in a natural population with no removals. This tumour, in the actual screen population, might be detected and removed before screening; we wish to calculate the probability $p$ of this detection. Let $\mu(y)$ be the probability density (with respect to tumour size) that a tumour is detected at size $y$. Then the probability of detection before the tumour reaches size $s$ is:

$$p = \int_0^s \mu(y)\,dy$$

We can estimate the value of this integral using the data for the control population. These data consist of the number $n_k$ of tumours detected between sizes $y_{k-1}$ and $y_k$, for each of the $m$ size categories, $k = 1, 2, \ldots, m$. The probability density $\mu$ in the $k_{th}$ size class is thus approximately:

$$\mu(y) \approx \frac{n_k}{n(y_k - y_{k-1})}$$

where $n = \sum_{k=1}^{m} n_k$ is the total number of tumours detected in the control population. The tumour of size $s$ will be on average approximately in the middle of its size category $k_s$. Thus, the above integral can be approximated as:

**Figure 1A** Three possible growth patterns of human primary breast cancer. For Gompertz growth $\alpha = 0.66$ and $k_0 = 19$ (Norton, 1988), which correspond to a limiting volume of 3100 ml. Fournier et al (1980), by analysing consecutive mammograms of 160 breast cancer patients, estimated that the mean volume doubling time of a tumour of 1.7 cm in diameter (2.6 ml in volume) is about 7 months. For exponential growth, this corresponds to $k = 1.2$ years$^{-1}$. For parabolic growth (power law growth with $\beta = 0.5$, equation 2), the parameter $k = 1.6$ was estimated from the same data. For all three models the initial tumour volume, $y_0$, is held to be a volume of a single cell. The tumour volume was calculated by assuming that primary breast cancer grows as a spheroid. **B** A semilogarithmic plot of the mass-specific growth rates vs tumour diameter for the discussed growth laws. The same parameters as in Figure 1a were used

$$p = \left[\sum_{k=1}^{k_s-1} \frac{n_k}{n(y_k - y_{k-1})}(y_k - y_{k-1})\right] + \frac{n_{k_s}}{n\left(y_{k-1} - y_{k_s}\right)} \cdot \frac{y_{k-1} - y_{k_s}}{2} = \left[\sum_{k=1}^{k_s-1} \frac{n_k}{n}\right] + \frac{n_{k_s}}{2n}$$

### Theoretical distributions of tumour sizes in populations

The growth rate of solid tumours depends on a multiplicity of factors, such as vascularity and nutrient supply, interactions with surrounding tissues, growth factors, regulation of apoptosis, and so on. These factors themselves vary with tumour size. Thus, the rate of growth of the tumour can be considered as a function of size. To express this in mathematical terms, let $y(t)$ denote the tumour mass at time $t$. Then the tumour will grow according to some differential equation of the form:

$$\frac{dy}{dt} = f(y) \tag{3}$$

where $f$ is some differentiable function of the tumour mass $y$.

We wish to derive the probability density $\varphi(y)$ that a tumour, randomly chosen from a certain size range and growing according to the differential equation (3), is of size $y$, assuming no removals due to treatment or death. It will also be assumed that the distribution of tumors, $\varphi(y)$, is stationary, i.e. the probability density of tumours is independent of time. This is reasonable for populations that are fairly stable demographically, and for which there has been no 'point event' (such as an acute exposure to radiation or other carcinogens) that would cause an unusually large number of tumours to be formed at about the same time.

Consider the population of tumours whose masses lie in the interval $y$ and $y + \Delta y$. Tumours are entering this population at the rate:

$$\varphi(y)\frac{dy}{dt} = \varphi(y)f(y)$$

they are leaving the population at the rate:

$$\varphi(y + \Delta y)f(y + \Delta y) = \varphi(y + \Delta y)\,(f(y) + f'(y)\Delta y) + o(\Delta y)$$

Equating these two quantities and rearranging gives:

$$\frac{\varphi(y + \Delta y) - \varphi(y)}{\Delta y} = -\varphi(y + \Delta y)\frac{f'(y)}{f(y)} + o(\Delta y) \tag{4}$$

so taking the limit as $\Delta y \rightarrow 0$ gives the differential equation:

$$\varphi'(y) = -\varphi(y)\frac{f'(y)}{f(y)} \tag{5}$$

Equation 5 has the general solution:

$$\varphi(y) = \frac{C}{f(y)} \tag{6}$$

where $C$ is a constant chosen to normalize the probability density to one. In the case of power law growth (equation 1), $f(y) = ky^{\beta}$, and hence

$$\varphi'(y) = \frac{-\beta\varphi(y)}{y} \tag{7}$$

Using equation 6, equation 7 becomes

$$\varphi(y) = \frac{C}{y^{\beta}} \tag{8}$$

Note that as this result does not depend on the tumour growth rate parameter $k$, it is valid even when (as is actually the case) $k$ varies in the population, provided the distribution of $k$ values is also stationary.

Gompertz growth satisfies the differential equation:

$$\frac{dy}{dt} = k_0 e^{-\alpha y} \tag{9}$$

where $k_0$ and $\alpha$ are constants. This equation can be transformed into the autonomous form of equation 3 with $f$ given by:

$$f(y) = -ry\ln(y/K) \tag{10}$$

where $K$ is the limiting size of the tumour and $r$ is a constant (Edelstein-Keshet, 1988). Inserting this into equation 6 gives:

$$\varphi(y) = \frac{C}{y(\ln K - \ln y)} \tag{11}$$

The logistic differential equation is:

$$\frac{dy}{dt} = r_0 y\left(1 - \frac{y}{K}\right) \tag{12}$$

where $r_0$ and $K$ are constants, representing the intrinsic growth rate and the limiting size of the tumour respectively. From equation 6 we have:

$$\varphi(y) = \frac{C}{y(K - y)} \tag{13}$$

The graphs of the theoretical distributions derived in equations 8, 11 and 13 with best fit of the two-county Swedish data are shown in Figure 2.

### RESULTS

We focused our analysis on the size distribution of tumours found in the first screen of the two-county Swedish mammography trial, which is one of the largest and most detailed studies of its kind (Tabar et al, 1992). Other published mammography screening trials (Thomas et al, 1984; Fagenberg et al, 1985; Burhenne et al, 1992; Peer et al, 1994; de Koning et al, 1995) are less detailed, but can provide collaborative information about the tumour size distribution (Table 1).

Our first aim was to reconstruct from the two-county Swedish mammography data the natural tumour size distribution in the population, i.e. what the size distribution would have been had there been no removals before the first screen. To this end, we employed the distribution of tumour sizes at detection in the two-county Swedish study's large corresponding control group. We reconstructed the natural tumour size distribution by estimating the probability, $p$, that a tumour of a given size category would have been detected without screening (see Methods), and then divided the number of tumours detected by mammography in each category by $1 - p$. We excluded from the analysis the smallest (< 1 cm) size category because of reduced mammography sensitivity in small tumours (Feig et al, 1977; Yaffe et al, 1993). As the probability of self-detection in the largest size category (> 5 cm) is close to 1, dividing by $1 - p$ would produce a number extremely sensitive to the exact value of $p$, and thus be unreliable; therefore, this size category was excluded as well (Table 1). We assumed that in the 1- to 5-cm range there is little variation in detection sensitivity (with the possible exception of the 1- to 1.5-cm category). Hence, we took the probability of detection in these size categories as constant.

© Cancer Research Campaign 1998

**Figure 2** The best fit of the theoretical density distributions of tumour size (equations 8, 11 and 13) to the reconstructed natural distribution estimated from the two-county Swedish data (Tabar et al, 1992) (▲). Results are presented on a log–log plot. A highly significant linear fit of the data was obtained with a slope $-\beta = -0.42$; ($r^2 = 0.97$). The Gompertz law was fitted using a limiting size of 3100 ml (Norton, 1988). The logistic growth was fitted using a limiting size of 1000 ml estimated by Spratt et al (1993)

**Figure 3** Estimation of mammography sensitivity in small tumours (0.5–1.5 cm). The theoretical density distribution of tumour size (assuming power law growth, equation 8) was fitted to the two-county Swedish data (in the 1.5–5.0 cm range) (▲) and subsequently was extrapolated to the smallest tumour range. The ratio between the theoretical and the empirical points can be readily converted to the relative probability of detection. The mammography in the 1–1.5 cm range detects at most 70% of the prevalent tumours. In the 0.5–1 cm range no more than 40% of the tumours are detected. Results are presented on a log–log scale

**Table 1** Size distribution of screened, control and reconstructed natural tumour populations, obtained from published breast cancer screening trials. Only the first screen data for tumours are used. The probability density (*P. density*) for a tumour between 1 and 5 cm in a natural population to be found in a particular size category is estimated. The results of linear regression of the logarithm of the natural tumour size distribution vs the logarithm of the relevant tumour volumes (linear slope $-\beta$, and the corresponding $r^2$) are presented. For those data sets where the number of data points was not sufficient to perform a regression analysis, only the linear slopes were calculated

| Source | Tumour size (cm)[a] | No. screen[b] | No. control[c] | *P. density*[d] | −β (line slope) | $r^2$ |
|---|---|---|---|---|---|---|
| Swedish two-county (11 year) (Tabar et al, 1992) | 0.1–1 | 100 | 50 | | −0.42 | 0.97 |
| | 1–1.4 | 112 | 107 | 0.53 | | |
| | 1.5–1.9 | 74 | 143 | 0.44 | | |
| | 2–2.9 | 57 | 216 | 0.26 | | |
| | 3–4.9 | 24 | 143 | 0.13 | | |
| | 5+ | 15 | 68 | | | |
| Total | | 382 | 727 | | | |
| Swedish two-county (6 years) (Fagerberg et al, 1985) | 0–1 | 87 | 32 | | −0.41 | |
| | 1.1–2 | 79 | 120 | 0.48 | | |
| | 2.1–5 | 32 | 103 | 0.17 | | |
| | 5.1+ | 5 | 17 | | | |
| Total | | 203 | 272 | | | |
| Guildford (Thomas et al, 1984) | 0–0.5 | 26 | | | −0.32 | 0.99 |
| | 0.6–1.5 | 17 | | 0.52 | | |
| | 1.6–2 | 9 | | 0.34 | | |
| | 2.1–5 | 15 | | 0.19 | | |
| | 5+ | 1 | | | | |
| Total | | 68 | | | | |
| Netherlands (de Koning et al, 1995) | 0–1 | 549 | 307 | | −0.43 | |
| | 1.1–2 | 834 | 1248 | 0.5 | | |
| | 2+ | 362 | 2051 | 0.17 | | |
| Total | | 1744 | 3606 | | | |
| Nijmegen (Peer et al, 1994) | 0–1 | 40 | 26 | | −0.54 | |
| | 1.1–2 | 92 | 101 | 0.54 | | |
| | 2+ | 36 | 215 | 0.1 | | |
| Total | | 168 | 342 | | | |
| British Columbia (Burhenne et al, 1992) | 0–1 | 19 | | | −0.38 | |
| | 1.1–2 | 10 | | 0.47 | | |
| | 2+ | 5 | | 0.18 | | |
| Total | | 34 | | | | |

[a]Tumour diameters as reported in the source articles. [b]Number of detected cancers in the group. [c]In data sets with no control population, the Swedish control incidence rates were used. [d]*P. density* is the reconstructed natural probability density for the tumour population between 1 and 5 cm (0.6 and 5 cm in Guildford).

We compared this empirical distribution with the theoretical distributions of primary tumour volumes corresponding to power, Gompertz and logistic growth laws (see Methods). Figure 2 displays the best-fit plots of these theoretical distributions to the reconstructed natural distribution obtained from data of the two-county Swedish trial (Tabar et al, 1992). The points calculated from the trial data lie nearly on a straight line with slope $-\beta = -0.42$ ($r^2 = 0.97$), indicating a power law growth function with $\beta = 0.42$. Note that these data are inconsistent with exponential growth (power law with $\beta = 1$), nor are they consistent with Gompertz or logistic growth laws with the accepted limiting sizes (Norton, 1988; Spratt et al, 1993). Most of the non-linearity in the two-county Swedish data is due to the density of the lowest size category (1–1.5 cm), where the sensitivity of the mammography may be less than for larger tumours. Excluding this point gives a slightly higher exponent ($\beta = 0.53$; $r^2 = 0.99$). Thus, the evidence indicates that primary breast cancer growth is parabolic (power law growth, $\beta \sim 0.5$).

### Verification of the result using independent screening trials

We verified our result by using data from other published mammography screening trials; only studies that contain sufficient information for analysis were included. Our analysis of these data, including an earlier report of the same Swedish study discussed above (Fagerberg et al, 1985), give consistent results: the data in all cases are compatible with power law growth, with $\beta$ between 0.32 and 0.55 (Table 1). The slopes of the UK (Thomas et al, 1984) and British Columbia (Burhenne et al, 1992) trials ($\beta = 0.32$ and $\beta = 0.38$) are even more contradictory to the Gompertz law. However, as these trials did not have their own control groups, they are less reliable. If Swedish women were more careful about regular self-examination, there would be more large tumours removed before screening compared with the British or the British Columbian studies. In such a case the probability of detection before screening, $p$, in the larger size classes would be overestimated by the use of the two-county Swedish control, so the slope would be underestimated. Note that in all the controlled studies the slope ranged between 0.41 and 0.54. It appears, then, that a control group in each screening trial is important for the use of this technique.

### DISCUSSION

Our results suggest that tumour size increases approximately as a quadratic function of time (i.e. parabolic growth). This is slower than exponential, but without the limiting asymptotic size suggested by sigmoidal growth models. Parabolic growth indicates a mass-specific growth rate that declines with the square root of tumour mass, as opposed to the constant mass-specific growth rate of exponential growth. Whereas the Gompertz and the logistic laws also predict a slowing mass-specific growth rate, these declines, using the parameters estimated in Norton (1988) and Spratt (1993), are less significant in the clinical size ranges than those predicted by parabolic growth (Figure 1B). This may imply that the response of breast cancer to chemotherapy may be different than would be suggested by the Norton–Simon model that assumes Gompertz growth (Norton and Simon, 1986).

There is substantial evidence at the cellular level of a decline in the mass-specific growth rate as tumours increase in size. Studies of the cytokinetics of both human breast cancer and experimental tumours show that the thymidine labeling index (TLI) declines in larger tumours, indicating that the fraction of cells that are actively growing is decreasing (Schiffer et al, 1979; Meyer and Coplin, 1988). Recent reports indicate that the vascular density of tumours may decline with growth (Holmgren et al, 1995). In such a case a significant fraction of tumour cells that lie too far from a capillary will be driven to a non-proliferating state or possibly even to death.

It should be emphasized that our method does not require knowledge of the absolute sensitivity of detection. Rather, in this work we made a simple and not unreasonable assumption that the sensitivity in the 1- to 5-cm size range is approximately constant. If independent measures of mammography sensitivity could be obtained [e.g. by comparing with magnetic resonance imaging (MRI)] it would be possible to use our method for estimating the tumour growth law for smaller size categories.

Alternatively, assuming that the parabolic growth law holds for the smaller size categories, the result of this study can be used for estimating the relative sensitivity of mammography in smaller tumours. This can be done by observing the deviation from linearity in these size categories in the log–log tumour natural size distribution plot. For example, it appears, by analysing data from the two-county Swedish trials, that mammography in the 1–1.5 cm range is about a third less sensitive than for larger tumours (Figure 3). By extrapolating the regression line to 0.5- to 1-cm range, we estimated that the relative sensitivity of mammography in this size range is about 40% (assuming $\beta = 0.42$) or about 30% (assuming $\beta = 0.53$) compared with larger tumours. This type of sensitivity analysis, combined with the power law for breast cancer growth, may help determine the optimal time period between screening mammography.

This study also may have implications for breast cancer cell kinetic parameter estimation. For instance, the tumour's potential doubling time and cell loss factor, which may be useful for dose calculation in radiotherapy, are calculated under the assumption of a constant cell cycle time and an exponential tumour growth, respectively (Steel, 1967, 1989; Bertuzzi et al, 1995). If, as our study suggests, tumours follow parabolic growth, it would be necessary instead to estimate the patient-specific growth constant, $k$ (equation 1), which is probably highly variable (Fournier et al, 1980).

Alternating chemotherapy regimens, proposed by Goldie and Coldman for minimizing the risk of drug resistance (Goldie and Coldman, 1979), have been the rationale of numerous anti-cancer protocols for the last 20 years. Our findings may imply an alternative strategy. If, as our results suggest, there is a significant decline in the percentage of actively dividing cells in large tumours (Figure 1B), these tumours would be less sensitive to cycle-specific therapies. Therefore, they may be better treated first with rather broader activity antineoplastic drugs, such as anthracyclines or alkylating agents. This may be an explanation for the observation that alternating the non-cell cycle-specific drug, doxorubicin, with CMF (cyclophosphamide, methotrexate, 5-fluorouracil, the last two drugs being cell cycle specific) is significantly inferior to a sequential chemotherapy protocol with doxorubicin as the first drug for high-risk (i.e. large tumour burden) breast cancer (Bonadonna et al, 1995).

Our results refer to the growth of untreated tumours only and their relevance for the growth patterns of tumours under treatment remains to be investigated. Nevertheless, it is interesting to note that the relative benefit of accelerated irradiation strategy (Corvo et al, 1995) may be explained in part by our results. If irradiated tumours are subject to a similar power law growth, according to which as

© Cancer Research Campaign 1998

tumours shrink under treatment their growth fraction increases, then the latter period of therapy should be more aggressive.

The optimal growth patterns of interacting cell assemblies have recently been shown to follow parabolic or other power laws (Drasdo et al, 1995). These theoretical results corroborate our analysis of clinical data, and imply that power growth law may have greater generality than just to mammary tumours. Our very preliminary analyses of thyroid cancer and renal cell carcinoma screening data suggest that the growth rate of these tumours may also follow a power law. More empirical evidence is needed to assess the universality of power law growth and its usefulness in the control of cancer.

### ACKNOWLEDGEMENTS

The authors wish to acknowledge A Bertuzzi, S Chaitchik and A Gandolfi for helpful discussion, M Kimmel, S Moss, L Norton and GS Steel, for critically reading the manuscript and R Ben-Shitrit for technical assistance. Our work was supported in part by the Chai Foundation and the Israel Cancer Association.

### REFERENCES

Bertuzzi A, Gandolfi A, Sinisgalli C and Starace G (1995) Estimation of cell cycle kinetic parameters by flow cytometry. In *Proceedings of the 4th International Conference on Mathematical Population Dynamics*, Arino O, Axelrod DE and Kimmel M (eds), pp. 167–180 World Scientific: Houston

Bonadonna G, Zambetti M and Valgussa P (1995) Sequential or alternating doxorubicin and CMF regimens in breast cancer with more than three positive nodes. Ten-year results. *JAMA* **273**: 542–547

Burhenne LJW, Hislop TG and Burhenne HJ (1992) The British Columbia mammography screening program: evaluation of the first 15 months. *AJR* **158**: 45–49

Corvo R, Giaretti W, Sanguineti G, Geido E, Orcchia R, Guenzi M, Margarino G, Bacigalupo A, Garaventa G, Barbieri M and Vitale V (1995) In vivo cell kinetics in head and neck squamous cell carcinomas predicts local control and helps guide radiotherapy regimen. *J Clin Oncol* **13**: 1843–1850

Crown J (1997) High dose chemotherapy of metastatic breast cancer: the end of the beginning? *Br J Cancer* **75**: 467–469

de Koning HG, Frachebound J, Boer R, Verbeek ALM, Collette HJA, Hendriks JHCL, van Ineveld BM, de Bruyn AE and van der Maas PJ (1995) Nation-wide breast cancer screening in the Netherlands: support for breast-cancer mortality reduction. *Br J Cancer Inst* **60**: 777–780

Dethlefsen LA, Prewitt JMS and Mendelsohn ML (1968) Analysis of tumor growth curves. *J Natl Cancer Inst* **40**: 389–405

Drasdo D, Kree R and McCaskill JS (1995) Monte Carlo approach to tissue-cell populations. *Phys Rev E* **52**: 6635–6657

Edelstein-Keshet L (1988) *Mathematical Models in Biology*, p. 217. McGraw-Hill: New York

Fagerberg G, Baldetorp L, Grontoft O, Lundstrom B, Manson JC and Nordenskjold B (1985) Effect of repeated mammographic screening on breast cancer stage distribution. *Oncology* **24**: 465–473

Feig SA, Shaber GS, Patchefsky A, Schwartz GO, Edeiken J, Libshitz HI, Nerlinger R, Curley RF and Wallace JD (1977) Analysis of clinically occult and mammographically occult breast tumours. *AJR* **128**: 403–408

Flehinger BJ, Kimmel M, Polyak T and Melamed M (1993) Screening for lung cancer. *Cancer* **72**(5): 1573–1580

Fletcher SW, Black W, Harris R, Rimer B and Shapiro S (1993) Report of an international workshop on screening for breast cancer. *J Natl Cancer Inst* **85**: 1644–1655

Fournier DV, Weber E, Hoeffken W, Bauer M, Kubli F and Barth V (1980) Growth rate of 147 mammary carcinomas. *Cancer* **45**: 2198–2207

Gershon-Cohen J, Berger M, Klickstein HS (1963) Roentgenography of breast cancer moderating concept of biological predeterminism. *Cancer* **16**: 961–964

Goldie JH and Coldman AJ (1979) Mathematic model for relating the drug sensitivity of tumors to their spontaneous mutation rate. *Cancer Treatment Rep* **63**: 1727–1733

Heuser L, Spratt J and Polk HC (1979) Growth rates of primary breast cancers. *Cancer* **43**: 1888–1894

Holmberg LH, Tabar L, Adami HO and Bergstrom R (1986) Survival in breast cancer diagnosed between mammographic screening examinations. *Lancet* **i**: 27–30

Holmgren L, O'Reilly MS and Folkman J (1995) Dormancy of micrometastases: balanced proliferation and apoptosis in the presence of angiogenesis suppression. *Nature Med* **1**: 149–153

Kimmel M and Flehinger BJ (1991) Nonparametric estimation of the size–metastasis relationship in solid cancer. *Biometrics* **47**: 987–1004

Laird AK (1965) Dynamics of tumor growth: comparison of growth rates and extrapolation of growth curve to one cell. *Br J Cancer* **20**: 278–291

Mendelsohn ML (1963) Cell proliferation and tumour growth. In *Cell Proliferation*, Lamberton LF and Fry RJM (eds), pp. 498–513. Blackwell Scientific Publications: London

Meyer JS and Coplin MD (1988) Thymidine labelling index, flow cytometric S-phase measurement, and DNA index in human tumors. *Am J Clin Pathol* **89**: 586–595

Norton L (1988) A Gompertzian model of human breast cancer growth. *Cancer Res* **48**: 7067–7071

Norton L and Simon R (1976) Measuring the course of gompertzian growth. *Nature* **264**: 542–545

Norton L and Simon R (1986) The Norton–Simon hypothesis revised. *Cancer Treat Rep* **10**: 163–169

Peer PG, Dijck JAAMV, Hendriks JHCL, Holland R and Verbeek ALM (1993) Age-dependent growth rate of primary breast cancer. *Cancer* **71**: 3547–3551

Peer PGM, Holland R, Hendriks JHCL, Mravunac M and Verbeek ALM (1994) Age-specific effectiveness of the Nijmegen population-based breast cancer-screening program: assessment of early indicators of screening effectiveness. *J Natl Cancer Inst* **86**: 436–441

Schiffer LM, Braunschweiger PG, Stragand JJ and Poulakos L (1979) The cell kinetics of human mammary cancers. *Cancer* **43**: 1707–1719

Spratt JA, von Fournier D, Spratt JS and Weber EE (1993) Decelerating growth and human breast cancer. *Cancer* **71**: 2013–2019

Steel GG (1967) Cell loss as a factor in the growth of human tumors. *Eur J Cancer* **3**: 381–387

Steel GG (1989) Cell proliferation kinetics in tumours. In *The Biological Basis of Radiotherapy*, 2nd edn, Steel GG, Adams GE and Horwich A (eds), pp. 77–88. Elsevier: Amsterdam.

Steel GG and Lamerton LF (1966) The growth rate of human tumours. *Br J Cancer* **20**: 74–86

Tabar L, Fagerberg G, Duffy S, Day NE, Gad A and Grontoft O (1992) Update of the Swedish two-county program of mammographic screening for breast cancer. *Radiol Clin North Am* **30**: 187–210

Tabar L, Fagerberg G, Chen HH, Duffy SW, Smart CR, Gad A and Smith RA (1995) Efficacy of breast cancer screening by age. New results from the Swedish Two-County Trial. *Cancer* **75**: 2507–2517

Thomas BA, Price JL, Boulter PS and Gibbs NM (1984) The first three years of the Guildford breast cancer project. *Cancer Res* **90**: 195–199

Xu JL and Prorok PC (1997) Nonparametric estimation of the solid cancer size and probability of presenting with metastasis at detection. *Biometrics* **53**: 579–591

Yaffe MJ, Jennings RJ, Fahrig R and Fewell TR (1993) X-ray spectral considerations for mammography. In *RSNA Scientific Assembly and Annual Meeting: Categorical Course in Physics: Technical Aspects of Breast Imaging*, Haus A and Yaffe M (eds) pp. 63–72. Radiological Society of North America: Oak Brook

© Cancer Research Campaign 1998